# Incidence and risk factors for perioperative hypotension during noncardiac surgery: A retrospective cohort study

**DOI:** 10.1097/MD.0000000000046451

**Published:** 2026-01-09

**Authors:** Zhilin Sun, Kaiyu Wang, Peining Ji

**Affiliations:** a Department of Anesthesiology, Liangxiang Hospital, Beijing, China; b Department of Anesthesiology, Maternal and Child Health Hospital of Fangshan District, Beijing, China.

**Keywords:** acute kidney injury, anesthesia modality, myocardial injury, noncardiac surgery, perioperative hypotension, risk factors

## Abstract

Perioperative hypotension (POH) is frequent in noncardiac surgery and is linked to acute kidney injury (AKI), myocardial injury after noncardiac surgery (MINS), and increased postoperative resource use. Previous studies have applied inconsistent POH definitions and risk models. This study aimed to determine the incidence of POH and identify independent risk factors, while assessing the robustness of findings across alternative thresholds. In this single-center retrospective cohort, 3000 adults (≥18 years) undergoing noncardiac surgery between April 2023 and December 2024 were analyzed using data from electronic medical and anesthesia information systems. POH was defined as intraoperative mean arterial pressure (MAP) <65 mm Hg lasting ≥5 minutes. Multivariable logistic regression identified independent predictors among prespecified covariates: American Society of Anesthesiologists (ASA) physical status, baseline MAP, surgical risk, emergency status, continuation of angiotensin-converting enzyme inhibitors/angiotensin receptor blockers (ACEI/ARB) on the day of surgery, operative duration, intraoperative vasoactive use, and anesthesia modality. Sensitivity analyses tested alternative MAP thresholds (<60, <65, and <70 mm Hg). POH occurred in 775 patients (25.8%). Incidence varied by anesthesia technique—76.6% for general, 75.4% for neuraxial, and 68.7% for peripheral nerve block—and by surgical risk: 70.3% (low), 75.0% (intermediate), and 79.4% (high). Independent predictors included ASA III–IV (odds ratio [OR] 1.71, 95% confidence interval 1.44–2.02), lower baseline MAP per 10-mm Hg decrease (OR 1.19, 1.10–1.28), high- (OR 1.65, 1.30–2.11) and intermediate-risk surgery (OR 1.30, 1.08–1.56), emergency surgery (OR 1.65, 1.30–2.08), continuation of ACEI/ARB (OR 1.33, 1.10–1.60), longer operative duration (per hour: OR 1.11, 1.04–1.19), and intraoperative vasoactive use (OR 1.26, 1.07–1.49). Neuraxial anesthesia versus general anesthesia was protective (OR 0.71, 0.58–0.87). POH was associated with higher incidences of AKI (30.2% vs 2.2%), MINS (27.6% vs 1.9%), and intensive care unit admission (52.9% vs 3.0%) (all *P* < .001). Sensitivity analyses across MAP <60/65/70 mm Hg confirmed consistent predictors. POH is common during noncardiac surgery and strongly associated with postoperative AKI, MINS, and intensive care unit admission. Major risk factors include higher ASA class, lower baseline MAP, higher surgical risk, emergency surgery, perioperative ACEI/ARB continuation, longer duration, and vasoactive use, while neuraxial anesthesia may reduce risk. These results support individualized hemodynamic optimization to mitigate POH and its adverse outcomes.

## 1. Introduction

Perioperative hypotension (POH) is frequent among patients undergoing noncardiac surgery and has been associated with higher risks of acute kidney injury (AKI), myocardial injury-including myocardial injury after noncardiac surgery (MINS)—and short-term mortality.^[1,2]^ Observational data indicate that even brief reductions in mean arterial pressure (MAP) to 55–65 mm Hg are linked to incrementally greater risks of AKI and myocardial injury, with longer hypotensive exposure conferring higher risk.^[3]^ Consensus statements likewise emphasize that intraoperative MAP values below approximately 60 to 70 mm Hg are potentially harmful and recommend prompt identification of underlying causes—such as vasodilation, hypovolemia, bradycardia, or low cardiac output-followed by targeted intervention.^[4,5]^ In parallel, postoperative myocardial injury has a robust association with 30-day mortality, underscoring the importance of hemodynamic stability and myocardial protection across the perioperative period.^[6,7]^

Despite broad agreement on the adverse associations, definitions and quantification of POH remain inconsistent across studies. Investigators have variously used absolute thresholds, relative declines from baseline, or time-integrated metrics (e.g., threshold × time area), leading to heterogeneity in reported incidence and risk estimates. Emerging evidence suggests that patients with higher baseline risk may experience excess AKI even within “milder” hypotensive ranges (e.g., MAP 55–59 mm Hg), highlighting the potential value of individualized thresholds that integrate both nadir and exposure duration.^[8]^ Perioperative blood pressure is further shaped by patient factors (e.g., American Society of Anesthesiologists Physical Status [ASA] physical status and comorbidities) and procedure-related factors (surgical risk, emergency status, blood loss, and operative time). Anesthetic modality and medication management-such as continuation versus withholding of angiotensin-converting enzyme inhibitors/angiotensin receptor blockers (ACEI/ARB) on the day of surgery—may also influence the occurrence and consequences of hypotension, as reflected in consensus recommendations and clinical practice guidance.^[3,5]^

Against this background, we conducted a single-center, real-world cohort study (April 2023 to December 2024) to estimate the incidence of POH among adults undergoing noncardiac surgery and to identify independent risk factors. We also evaluated the associations between POH and key postoperative outcomes—AKI, MINS, intensive care unit (ICU) admission, and length of stay—and performed sensitivity analyses using alternative thresholds (MAP <60, <65, and <70 mm Hg) to test the robustness of our findings. We hypothesized that higher ASA class, lower baseline MAP, higher surgical risk, emergency presentation, continuation of ACEI/ARB on the day of surgery, longer operative duration, and intraoperative vasoactive administration would be independently associated with POH, and that POH would correlate with increased risks of adverse postoperative outcomes.

## 2. Materials and methods

### 2.1. Study design

This study was approved by the Ethics Committee of Liangxiang Hospital (approval no. LXH-2023-012), with the requirement for informed consent waived due to its retrospective design. Consecutive adult patients undergoing noncardiac surgery from April 2023 to December 2024 were included. Data were de-identified and obtained from the institutional electronic medical record and anesthesia information system.

### 2.2. Study population: inclusion and exclusion criteria

*Inclusion:* age ≥ 18 years; noncardiac surgery (elective or emergency) during the study window; anesthesia and perioperative monitoring completed in-house with complete anesthesia records.

*Exclusion:* cardiac surgery or procedures with cardiopulmonary bypass; craniotomy, major trauma resuscitation, or other extreme scenarios with materially different hemodynamic goals; missing anesthesia records or non-reconstructable intraoperative BP series; procedures with local anesthesia/analgesia only. If multiple operations occurred for a patient, only the first eligible procedure was included.

### 2.3. Data sources and variables

Demographics, preoperative assessment, intraoperative processes, and postoperative outcomes were integrated from institutional systems. Key variables:

*Demographics/baseline:* age, sex, body mass index (BMI), ASA physical status, comorbidities (hypertension, coronary artery disease, diabetes, chronic kidney disease, and chronic obstructive pulmonary disease), and revised cardiac risk index.

*Preoperative hemodynamics:* baseline MAP (most recent stable noninvasive reading or ward/clinic value).

*Surgical/anesthetic information:* surgical specialty and risk (low/intermediate/high), emergency status, anesthetic modality (general anesthesia, neuraxial anesthesia, and peripheral nerve block), operative duration (h), estimated blood loss (mL), crystalloid volume (mL), intraoperative vasoactive use, arterial line placement, and day-of-surgery ACEI/ARB continuation (morning or same-day dose before operating room entry).

*BP time series:* BP time series: Intraoperative MAP data were extracted directly from anesthesia machines and patient monitors. For patients with arterial lines, invasive MAP values were recorded continuously at beat-to-beat frequency. For noninvasive monitoring, automated oscillometric measurements were typically obtained every 3 to 5 minutes, with some cases set at 1- to 2-minute intervals during high-risk phases. All MAP data underwent automated and manual quality control: implausible spikes or physiologically inconsistent values were identified by logical range and temporal checks (e.g., sudden changes >30 mm Hg within <1 minute) and excluded. For analysis, MAP readings were time-aligned and interpolated to a 1-minute resolution (or the finest available) to standardize exposure estimates. Sensitivity analyses confirmed that using longer noninvasive BP intervals did not materially alter the direction or significance of key associations.

### 2.4. Postoperative outcomes

Endpoints included AKI within 7 days (per institutional labs and renal function changes), MINS based on the institutional biomarker strategy (peak and trajectory within 72 hours), ICU admission, and length of stay (calendar days from surgery to discharge).

### 2.5. Exposure definition

*Primary POH definition:* intraoperative MAP < 65 mm Hg with cumulative duration ≥5 minutes, allowing summation across episodes. Isolated outliers were screened by neighborhood comparison with predefined rules.

*Sensitivity definitions:* repeat the primary analyses with MAP < 60 mm Hg and MAP < 70 mm Hg. For exploratory comparison, time-under-threshold metrics were also recorded.

### 2.6. Outcomes

*Primary outcome:* occurrence of POH (primary definition).

*Secondary outcomes:* AKI, MINS, ICU admission, and length of stay. All were obtained from objective records; no additional follow-up was required.

### 2.7. Covariates and confounding

*Prespecified covariates (clinical a priori and literature-based):* age, sex, BMI, ASA (III–IV vs I–II), baseline MAP, surgical risk (intermediate/high vs low), emergency status, anesthetic modality, day-of-surgery ACEI/ARB continuation, operative duration (h), blood loss (>500 mL), and intraoperative vasoactive use. No univariable screening was used to avoid data-driven bias.

*Sample size, missing data, and bias control:* Convenience cluster sample (all eligible cases); no a priori calculation. Model stability ensured by EPV ≥ 10. Continuous variables underwent plausibility and outlier checks. If overall missingness was <5%, complete-case analysis was used; for any covariate with ≥5% missingness, multiple imputation by chained equations (m = 5) was applied and combined via Rubin rules as a sensitivity comparison.

### 2.8. Statistical analysis

*Descriptive:* continuous variables as mean ± standard deviation; categorical as n (%). Groups by POH status were compared using Welch *t* test (2-sided) and Pearson’s *χ*^2^ test.

*Multivariable model:* logistic regression with POH (yes/no) as the dependent variable. Scaling for interpretability: age (per 10 years), BMI (per 5 kg/m^2^), baseline MAP (per 10-mm Hg decrease), operative duration (per 1 hour). Surgical risk encoded with dummies (intermediate/high vs low); general anesthesia as the reference for anesthetic modality. Report adjusted odds ratios (ORs) with 95% confidence intervals (CIs).

*Multicollinearity/performance:* variance inflation factor <5 accepted. Discrimination via area under receiver operating characteristic curve; calibration via Hosmer–Lemeshow/calibration plots.

*Sensitivity analyses:* repeat the main model at MAP <60 and <70 mm Hg thresholds; where used, refit in multiple imputation by chained equations datasets and compare with complete-case estimates.

*Subgroup/exploratory:* stratify incidence by anesthetic modality and surgical risk; explore associations between POH and AKI/MINS/ICU/length of stay using *χ*^2^/*t* tests, presented in text or supplements as appropriate.

*Thresholds/software:* 2-sided *P* < .05 deemed significant; *P* values to 3 decimals (*P* < .001 as “<.001”); means/standard deviation and percentages to 2 decimals. Analyses were performed in R or Stata with figures adhering to journal standards.

## 3. Results

### 3.1. Study population and overall incidence

A total of 3000 noncardiac surgical patients were included; 775 (25.83%) experienced POH. Baseline characteristics are summarized in Table 1. In aggregate comparisons, higher ASA class, lower baseline MAP, emergency surgery, longer operative duration, and continuation of ACEI/ARB on the day of surgery were associated with a greater likelihood of POH. Distributions of multi-level variables (e.g., anesthetic modality and surgical risk) also differed between groups (Table 1).

**Table 1:** Baseline characteristics by POH status (total cohort: n = 3000; POH: n = 775; no POH: n = 2225).

Variable	Overall	POH	No POH	*t*/*χ*^2^	*P* value
Age, yr	62.37 ± 11.79	61.96 ± 11.72	62.51 ± 11.81	1.108	.268
BMI, kg/m²	25.95 ± 4.61	26.24 ± 4.62	25.85 ± 4.60	-2.043	.041
Baseline MAP, mm Hg	92.14 ± 10.97	93.65 ± 10.77	91.61 ± 10.99	-4.509	<.001
Surgery duration, h	2.38 ± 1.34	2.25 ± 1.18	2.43 ± 1.39	3.570	<.001
Estimated blood loss, mL	303.86 ± 214.92	293.37 ± 212.02	307.51 ± 215.85	1.592	.112
Crystalloid volume, mL	1694.00 ± 617.02	1702.97 ± 615.01	1690.87 ± 617.83	-0.471	.638
ASA 1	239 (7.97%)	32 (4.13%)	160(9.30)	42.473	<.001
ASA 2	979 (32.63%)	308 (39.74%)	671 (30.16%)		
ASA 3	1303 (43.43%)	301 (38.84%)	1002 (45.03%)		
ASA 4	479 (15.97%)	87 (11.23%)	392 (17.62%)		
Risk low	1083 (36.10%)	322 (41.55%)	761 (34.20%)	17.531	<.001
Risk intermediate	1325 (44.17%)	331 (42.71%)	994 (44.67%)		
Risk high	592 (19.73%)	122 (15.74%)	470 (21.12%)		
General anesthesia	2115 (70.50%)	520 (67.10%)	1595 (71.69%)	12.140	.002
Neuraxial anesthesia	603 (20.10%)	189 (24.39%)	414 (18.61%)	11.958	<.001
Peripheral nerve block	282 (9.40%)	66 (8.52%)	216 (9.71%)	0.959	.328
Male	1592 (53.07%)	402 (51.87%)	1190 (53.48%)	0.537	.464
Emergency = yes	563 (18.77%)	105 (13.55%)	458 (20.58%)	18.206	<.001
ACEI/ARB continued on DOS = yes	938 (31.27%)	207 (26.71%)	731 (32.85%)	9.8130	.002
Vasopressor use = yes	1697 (56.57%)	406 (52.39%)	1291 (58.02%)	7.202	.007
Arterial line = yes	1110 (37.00%)	254 (32.77%)	856 (38.47%)	7.763	.005
Hypertension = yes	1457 (48.57%)	392 (50.58%)	1065 (47.87%)	1.590	.207
Coronary artery disease = yes	521 (17.37%)	144 (18.58%)	377 (16.94%)	0.962	.327
Diabetes mellitus = yes	639 (21.30%)	167 (21.55%)	472 (21.21%)	0.021	.885
Chronic kidney disease = yes	318 (10.60%)	79 (10.19%)	239 (10.74%)	0.129	.720
COPD = yes	319 (10.63%)	72 (9.29%)	247 (11.10%)	1.797	.180

ACEI/ARB = angiotensin-converting enzyme inhibitors/angiotensin receptor blockers, ASA = American Society of Anesthesiologists, BMI = body mass index, DOS = day of surgery, MAP = mean arterial pressure, POH = perioperative hypotension.

### 3.2. Stratified incidence

**Figure 1.**
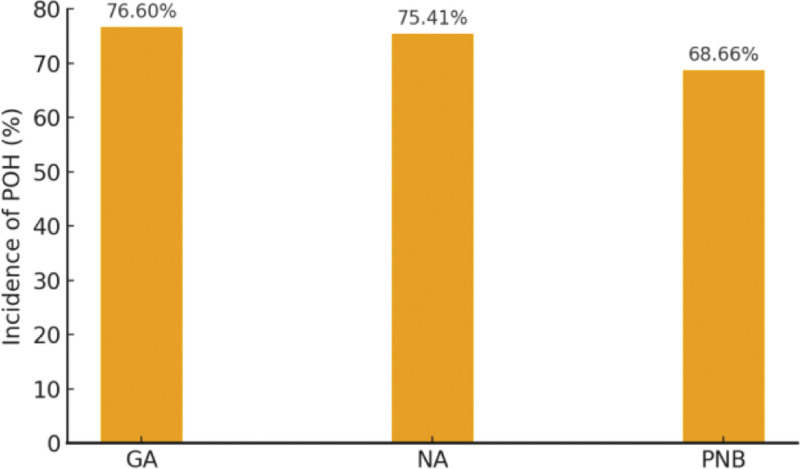
Incidence of POH by anesthesia type. POH = perioperative hypotension.

**Figure 2.**
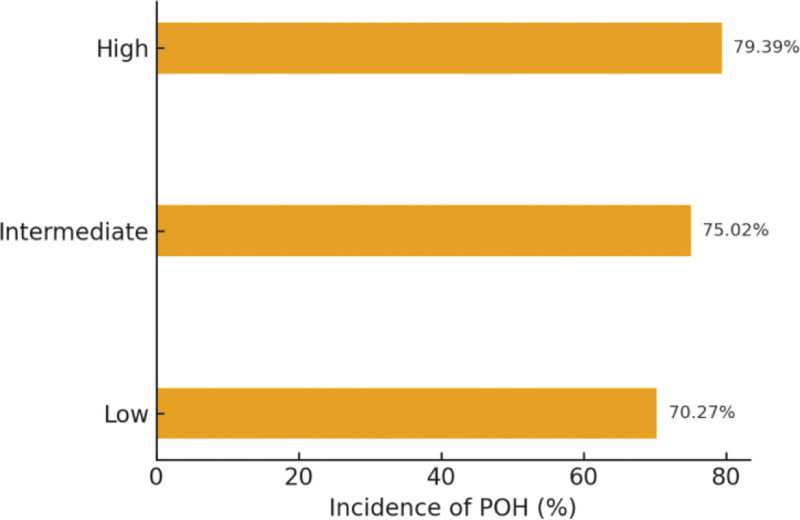
Incidence of POH by surgical risk category. POH = perioperative hypotension.

By anesthetic modality (Fig. 1), the incidence of POH was 76.60% with general anesthesia, 75.41% with neuraxial anesthesia, and 68.66% with peripheral nerve block. By surgical risk tier (Fig. 2), incidence was 70.27% for low-risk, 75.02% for intermediate-risk, and 79.39% for high-risk procedures.

### 3.3. Univariable and multivariable risk factors

In univariable comparisons (Table 2), several factors correlated with POH; in the multivariable logistic model, independent associations persisted for ASA III–IV (OR 1.71, 95% CI 1.44–2.02, *P* < .001), lower baseline MAP per 10-mm Hg decrement (OR 1.19, 95% CI 1.10–1.28, *P* < .001), surgical risk-high (OR 1.65, 95% CI 1.30–2.11, *P* < .001) and intermediate (OR 1.30, 95% CI 1.08–1.56, *P* = .006)—emergency surgery (OR 1.65, 95% CI 1.30–2.08, *P* < .001), day-of-surgery ACEI/ARB continuation (OR 1.33, 95% CI 1.10–1.60, *P* = .003), longer operative duration per hour (OR 1.11, 95% CI 1.04–1.19, *P* = .002), and intraoperative vasoactive use (OR 1.26, 95% CI 1.07–1.49, *P* = .007); neuraxial anesthesia versus general anesthesia showed a protective association (OR 0.71, 95% CI 0.58–0.87, *P* = .001), whereas age (*P* = .337), male sex (*P* = .434), BMI (*P* = .084), peripheral nerve block versus general anesthesia (*P* = .844), and blood loss > 500 mL (*P* = .432) were not significant (Table 3).

**Table 2:** Univariate logistic regression for POH (n = 3000).

Predictor	OR	95% CI	*P* value
Age (per 10 yr)	1.04	0.97–1.11	.270
Male	1.07	0.91–1.26	.439
BMI (per 5 kg/m^2^)	0.91	0.83–1.00	.041
ASA III–IV	1.67	1.42–1.97	<.001
Baseline MAP (per 10 mm Hg ↓)	1.19	1.10–1.28	<.001
High-risk surgery	1.43	1.15–1.78	.001
Intermediate-risk surgery	1.08	0.92–1.28	.343
Emergency surgery	1.65	1.31–2.08	<.001
Neuraxial versus GA	0.71	0.58–0.86	.001
Peripheral nerve block versus GA	1.15	0.87–1.54	.328
ACEI/ARB continued on DOS	1.34	1.12–1.61	.002
Surgery duration (per h)	1.11	1.04–1.19	.001
>500 mL blood loss	1.11	0.89–1.39	.361
Vasopressor use	1.26	1.07–1.48	.006

ACEI/ARB = angiotensin-converting enzyme inhibitors/angiotensin receptor blockers, ASA = American Society of Anesthesiologists, BMI = body mass index, CI = confidence interval, DOS = day of surgery, GA = general anesthesia, MAP = mean arterial pressure, OR = odds ratio, POH = perioperative hypotension.

**Table 3:** Multivariable logistic regression for POH (n = 3000).

Predictor	OR	CI	*P* value
Age (per 10 yr)	1.04	0.96–1.11	.337
Male	1.07	0.90–1.26	.434
BMI (per 5 kg/m²)	0.92	0.84–1.01	.084
ASA III–IV	1.71	1.44–2.02	<.001
Baseline MAP (per 10 mm Hg ↓)	1.19	1.10–1.28	<.001
High-risk surgery	1.65	1.30–2.11	<.001
Intermediate-risk surgery	1.30	1.08–1.56	.006
Emergency surgery	1.65	1.30–2.08	<.001
Neuraxial anesthesia versus GA	0.71	0.58–0.87	.001
Peripheral nerve block versus GA	1.03	0.76–1.39	.844
ACEI/ARB continued on DOS	1.33	1.10–1.60	.003
Surgery duration (per h)	1.11	1.04–1.19	.002
>500 mL blood loss	1.10	0.87–1.38	.432
Vasopressor use	1.26	1.07–1.49	.007

ACEI/ARB = angiotensin-converting enzyme inhibitors/angiotensin receptor blockers, ASA = American Society of Anesthesiologists, BMI = body mass index, CI = confidence interval, DOS = day of surgery, GA = general anesthesia, MAP = mean arterial pressure, OR = odds ratio, POH = perioperative hypotension.

### 3.4. Postoperative outcomes

Relative to the non-POH group, the POH group had significantly higher rates of AKI (30.19% vs 2.20%, *χ*^2^ = 527.136, *P* < .001), MINS (27.61% vs 1.93%, *χ*^2^ = 483.944, *P* < .001), and ICU admission (52.90% vs 2.97%, *χ*^2^ = 1073.743, *P* < .001) (Table 4).

**Table 4:** Postoperative outcomes by POH (total cohort: n = 3000; POH: n = 775; no POH: n = 2225).

Outcome	POH	No POH	*t*/*χ*^2^	*P* value
Acute kidney injury	234 (30.19%)	49 (2.20%)	527.136	<.001
Myocardial injury	214 (27.61%)	43 (1.93%)	483.944	<.001
ICU admission	410 (52.90%)	66 (2.97%)	1073.743	<.001

ICU = intensive care unit, POH = perioperative hypotension.

### 3.5. Sensitivity analyses

**Figure 3.**
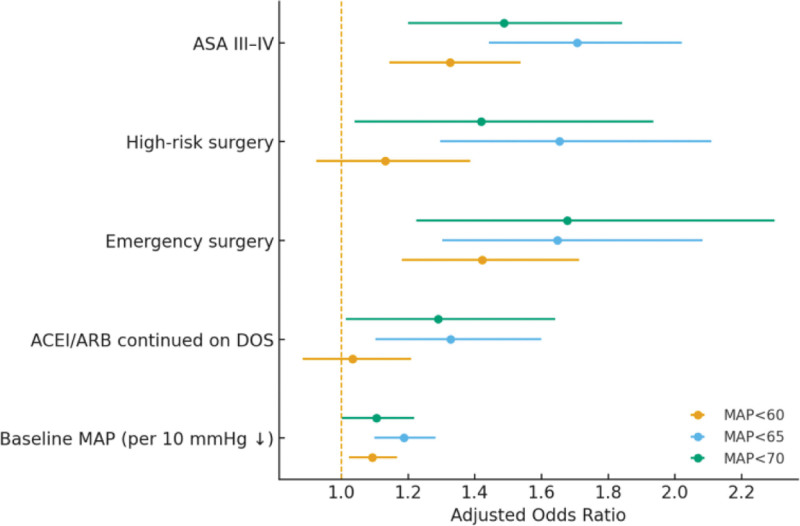
Stability of key predictors under alternative POH definitions. POH = perioperative hypotension.

Repeating the multivariable modeling under alternative POH thresholds (MAP <60, <65, and <70 mm Hg) yielded broadly consistent directions and statistical significance for key predictors (Table 5 and Fig. 3), supporting the robustness of the findings.

**Table 5:** Sensitivity analysis across alternative POH definitions (n = 3000).

Predictor	Definition	OR (95% CI)	*P* value
ASA III–IV	MAP < 60	1.33 (1.14–1.54)	<.001
ASA III–IV	MAP < 65	1.71 (1.44–2.02)	<.001
ASA III–IV	MAP < 70	1.49 (1.20–1.84)	<.001
High-risk surgery	MAP < 60	1.13 (0.93–1.39)	.226
High-risk surgery	MAP < 65	1.65 (1.30–2.11)	<.001
High-risk surgery	MAP < 70	1.42 (1.04–1.94)	.027
Emergency surgery	MAP < 60	1.42 (1.18–1.71)	<.001
Emergency surgery	MAP < 65	1.65 (1.30–2.08)	<.001
Emergency surgery	MAP < 70	1.68 (1.23–2.30)	.001
ACEI/ARB continued on DOS	MAP < 60	1.03 (0.88–1.21)	.670
ACEI/ARB continued on DOS	MAP < 65	1.33 (1.10–1.60)	.003
ACEI/ARB continued on DOS	MAP < 70	1.29 (1.01–1.64)	.038
Baseline MAP (per 10 mm Hg ↓)	MAP < 60	1.09 (1.02–1.17)	.008
Baseline MAP (per 10 mm Hg ↓)	MAP < 65	1.19 (1.10–1.28)	<.001
Baseline MAP (per 10 mm Hg ↓)	MAP < 70	1.11 (1.00–1.22)	.046

ACEI/ARB = angiotensin-converting enzyme inhibitors/angiotensin receptor blockers, ASA = American Society of Anesthesiologists, DOS = day of surgery, MAP = mean arterial pressure, POH = perioperative hypotension.

## 4. Discussion

In this single-center retrospective cohort (April 2023–December 2024), we observed a high incidence of POH (25.83%) among noncardiac surgical patients, with POH associated with greater risks of AKI, MINS, increased ICU utilization, and prolonged length of stay. In multivariable analyses, ASA III–IV, lower baseline MAP, higher surgical risk (intermediate/high), emergency presentation, day-of-surgery ACEI/ARB continuation, longer operative duration, and intraoperative vasoactive exposure were independently associated with POH, whereas neuraxial anesthesia (vs general anesthesia) showed a protective association. These findings support a blood-pressure management paradigm anchored on baseline risk and baseline hemodynamics, and highlight the influence of medication and anesthetic strategies on POH risk.

Consistent with prior evidence, our results align with a dose–response relationship between hypotensive “depth × time” burden and organ injury. Large retrospective and multicenter analyses have linked MAP below roughly 60–70 mm Hg—and longer exposure–to higher risks of AKI, myocardial injury, and mortality, which underpins consensus recommendations to avoid prolonged excursions in this range.^[9–11]^ Our sensitivity analyses across MAP <60/<65/<70 mm Hg reproduced the same directions of effect for key predictors, reinforcing this concept. Recent stratified work further suggests that depth may contribute more than duration, such that briefer but deeper hypotension can be more injurious than longer periods of mild hypotension.^[12]^ This is concordant with our observation that lower baseline MAP and longer surgery were associated with POH, emphasizing the need to prioritize prevention of profound nadirs while monitoring cumulative hypotension burden.

With respect to intervention strategies, the INPRESS randomized trial demonstrated that individualized blood-pressure targets-maintaining intraoperative systolic pressure within ±10% of each patient’s resting value-reduced postoperative organ dysfunction compared with standard care.^[13]^ Together with our finding that POH clusters in high-risk groups (higher ASA, high-risk procedures, and emergencies), these data support baseline-referenced targeting and continuous monitoring in vulnerable patients. Regarding perioperative ACEI/ARB management, multinational cohort analyses (e.g., VISION) suggest that withholding therapy within 24 hours before surgery is associated with fewer deaths/major vascular events and less intraoperative hypotension.^[14]^ Our data similarly link day-of-surgery ACEI/ARB continuation with POH, although high-quality trials are still needed to define candidates and timing for temporary discontinuation.

The salience of MINS was again evident in our cohort, where MINS occurred more often with POH. Large studies show that early postoperative high-sensitivity troponin elevations within 3 days strongly correlate with 30-day mortality—even without classic ischemic symptoms—underscoring the centrality of hemodynamic stability and myocardial protection across the perioperative course.^[15,16]^ Importantly, POH likely reflects multiple endotypes-including hypovolemia, vasodilation, pump failure, and drug-related mechanisms. Retrospective clustering analyses have identified distinct hypotension endotypes, suggesting that etiology-directed therapy is preferable to uniform reactive dosing.^[17]^ The observed association between intraoperative vasoactive use and POH in our study may reflect corrective treatment after POH onset rather than proactive prevention, emphasizing the value of early endotype recognition and preemptive, targeted management.

Methodologically, definitions and quantification of hypotension remain heterogeneous across the literature. Studies variably adopt absolute thresholds, relative declines, or time-integrated metrics (e.g., area under threshold), which can shift effect estimates and comparability.^[18]^ We therefore reported a unified primary POH definition and complemented it with threshold-based sensitivity analyses (MAP <60/<65/<70 mm Hg) to enhance robustness and comparability.

For high-risk populations-such as ASA III–IV, higher surgical risk, or emergency cases—we recommend: individualized MAP targets anchored to baseline pressure, with explicit priority to avoid profound nadirs; continuous or high-frequency monitoring to capture the “depth × time” burden; etiology-directed titration of volume, vasoactive agents, and anesthetic depth; and cautious perioperative ACEI/ARB strategies (consider preoperative withholding when no compelling indication exists, and evidence-based resumption postoperatively), embedded within institutional pathways.

Strengths of this study include its relatively large sample size, a standardized primary POH definition with multi-threshold sensitivity analyses, and prespecified covariate adjustment demonstrating internally consistent results. Limitations include the single-center retrospective design with potential residual confounding and misclassification; variable BP sampling resolution and monitoring modality (with vs without arterial line), which may underestimate brief episodes of deep hypotension; potential indication/selection bias around ACEI/ARB and anesthetic modality; and generalizability that warrants multicenter validation. Future prospective and randomized studies should test individualized MAP targets, endotype-guided interventions, and ACEI/ARB management strategies on hard outcomes such as AKI and MINS.

## 5. Conclusions

In this single-center retrospective cohort, perioperative hypotension (POH) was common during noncardiac surgery and was associated with increased risks of AKI, MINS, and ICU admission. Multivariable analyses identified ASA III–IV status, lower baseline MAP, higher surgical risk, emergency surgery, continuation of ACEI/ARB on the day of surgery, longer operative duration, and intraoperative vasoactive exposure as independent risk factors, whereas neuraxial anesthesia showed a protective association compared with general anesthesia. Results remained consistent across alternative POH thresholds (MAP <60/65/70 mm Hg), supporting robustness. These findings underscore the clinical importance of individualized intraoperative blood pressure management and tailored anesthetic strategies to mitigate postoperative organ injury and improve perioperative outcomes.

## Author contributions

**Conceptualization:** Zhilin Sun, Kaiyu Wang, Peining Ji.

**Data curation:** Zhilin Sun, Kaiyu Wang, Peining Ji.

**Formal analysis:** Zhilin Sun, Kaiyu Wang, Peining Ji.

**Funding acquisition:** Zhilin Sun.

**Investigation:** Zhilin Sun, Peining Ji.

**Writing – original draft:** Zhilin Sun, Kaiyu Wang, Peining Ji.

**Writing – review & editing:** Zhilin Sun, Kaiyu Wang, Peining Ji.
